# Statistical analysis of crash data and impact of COVID-19 on road crashes in Maharashtra, India

**DOI:** 10.1186/s44147-023-00194-9

**Published:** 2023-04-11

**Authors:** Krantikumar V. Mhetre, Aruna D. Thube

**Affiliations:** Civil Engineering Department, COEP Tech University (Formerly College of Engineering Pune), Pune, Maharashtra India

**Keywords:** Road safety, Lockdown, COVID-19, Crash, Data, Analysis

## Abstract

This study analyzes crash data from 2016 to 2020 on a National Highway in Maharashtra, India. The impact of the COVID-19 lockdown on the road crashes of the study area is presented, and recommendations to improve road safety are proposed. The crash data is collected from the “National Highways Authority of India, Kolhapur” from 2016 to 2020, and the information is classified into three scenarios: Before Lockdown, After Lockdown, and Strict Lockdown. The crash data is analyzed under three scenarios for seven different classifications followed by their sub-classifications. The time-wise analysis of crash data is performed in four-time slots, namely 00:00–05:59 AM, 06:00–11:59 AM, 12:00–17:59 PM, and 18:00–23:59 PM. The season-wise analysis of crash data is performed in three seasons: Summer, Monsoon, and Winter.

The crashes that occurred on 2-lane-straight roads having T-junction are more than 90% in all three scenarios. The significant factors responsible for crashes are “Head-on collision,” “Vehicle out of control,” and “Overspeeding.” Most crashes (more than 36%) occurred between 12:00 and 17:59 PM and in the Summer season (more than 42%) in all three scenarios. The crashes in the COVID-19 “Strict Lockdown” scenario witnessed a fall of 254.55% compared to 2019 and 2018. Surprisingly, there was a rise of 137.5% and a fall of 127.27% in crashes of the COVID-19 2020 “Strict Lockdown” scenario, compared to 2017 and 2016, respectively. The crashes under the sub-classifications “Right angle collision” and “Fatal” increased in 2020 compared to the previous 4 years due to the impact of COVID-19.

## Introduction

A lot of parameters are responsible for causing road crashes, like driving under the influence, overspeeding, poor road geometry, weather conditions, unavailability of traffic signs and signals, and defective vehicles. All these parameters cannot be treated by a single solution and therefore demands the engagement of multidisciplinary researchers and scientists in road crash studies and their minimization. In addition to this, the public participation is the major key factor to improve the road safety.

### Literature review

According to the “Ministry of Road Transport & Highways” report on “Road Accidents in India-2019” [6], India ranked first out of 199 countries in the fatalities caused due to road crashes. In the third global road safety conference held in Stockholm, Sweden, in 2020, all the countries promised to reduce the deaths caused due to road crashes by 50% by 2030 [8]. India accounts for 11% of the world’s road crash-related deaths. In 2019, India witnessed 449,002 road crashes, including 151,113 fatalities and 451,361 injuries.

The history of research works in the area of road safety started a long ago. Smeed [22] commented upon the statistics of road safety among various countries based on population and number of vehicles. Emenalo et al. [1] analyzed road traffic crash data in Zambia. The authors observed an increase of 103% in number of vehicles, 170% in road crash fatalities, and 194% in number of road crashes from 1964 to 1974. Landge et al. [4] developed a methodology for achieving road safety through community participation, which is an essential support in addition to engineering solutions and strict law enforcement. Rajaraman et al. [16] presented the data analysis of live crash records with conclusions and recommendations to reduce crashes of NH-45 throughout the 60-km length in Tamil Nadu, India. The study revealed that 58% of crashes were caused due to Head-on and Rear-end collisions involving heavy vehicles. Ponnaluri [13] analyzed the fatality rates in Andhra Pradesh (AP), India, from 2001 to 2009 and identified that the urban-to-rural share was 40%:60%. The authors have suggested developing a crash recording system, emergency response, and capacity building.

Gourav and Sachdeva [2] analyzed the crash data on NH-1 from 2007 to 2010 for a 50-km length in India. The study showed that 46% of crashes were due to overspeeding or driver’s fault, 42% involved heavy vehicles, and day-time crashes dominated over night-time crashes on NH-1. Mannering et al. [5] presented different statistical approaches available to deal with unobserved heterogeneity and concluded with a summary for future methodological works. Wegman [26] stated that 90% of all crashes occur in low- and middle-income countries. The author concluded that the increase in road crashes is notable unless road safety in low- and middle-income countries is improved. Singh [21] presented an analysis of road crashes in India and observed that the distribution of road crashes and injuries varied as per age, gender, month, and time. The author concluded that road crash fatalities in India by 2025 are likely to make up to 250,000 per year. Pandey and Pandey [10] studied road crash statistics in India and observed that the road crash is one of the four main reasons of death in India. Authors suggested the remedial measures be followed on various categories of roads in India to achieve road safety. Rabbani et al. [15] presented the statistical analysis of crash data in Peshawar, Pakistan, and stated the importance of crash data analysis to achieve road safety. Authors have found that the majority of crashes took place in daytime, and the 30–45 years age group was involved in majority of the road crashes.

### COVID-19 background

According to the “Ministry of Road Transport & Highways” report on “Road Accidents in India-2020” [7], India witnessed 366,138 road crashes, including 131,714 fatalities and 348,279 injuries. The annual road safety report by the “International Transport Forum” [3] presented recent road safety statistics of 42 countries belonging to the “International Road Traffic and Accident Database” (IRTAD) group. The effect of imposed lockdown due to the COVID-19 pandemic on road crash fatalities decreased by up to 80%. Countries like Denmark, the Netherlands, and Sweden have found an increase in road crash deaths by 9%, 6%, and 6%, respectively. As per European Transport Safety Council’s PIN briefing [12], out of 25 European countries, 19 witnessed a reduction in road crashes in April 2020 compared to April 2019, 2018, and 2017. The road crash fatalities reduction was 3% for 2018–2019, whereas 24% over the decade 2010–2019. The highest reduction in road crash fatalities was 84% in Italy and more than 59% in Belgium, Spain, France, and Greece.

Qureshi et al. [14] studied the effect of mandated societal lockdown on road traffic crashes in Missouri. Authors have found a significant reduction in road traffic crashes with minor injuries and an increase in crashes with fatalities, whereas a significant increase in road traffic crashes with minor injuries is observed after the end of mandatory societal lockdown. Yasin et al. [27] reviewed the effects of the COVID-19 pandemic on road traffic crashes at the global level. The authors have seen reduced road crash fatalities in 32 out of 36 countries compared to April 2019, with a decrease of 50% or more in 12 countries, 25–49% in 14 countries, and less than 25% in 6 countries. In contrast, road crash fatalities increased in the remaining four countries.

Sekadakis et al. [18] studied the impact of COVID-19 on road traffic crashes, fatalities, and injuries in Greece from January 2010 to August 2020. Authors observed a decrease in road traffic crashes, fatalities, and injuries due to the sharp reduction in traffic volume. Also, rates of fatalities and slightly injured are found to be increased in the lockdown period. Authors concluded that the worst performance in road safety is observed. Vanlaar et al. [25] studied the impact of COVID-19 on the risky driving behaviors of drivers between the USA and Canada. Authors found that the USA drivers had a higher percentage of risky driving behavior as compared to Canadian drivers in the COVID-19 lockdown period. In addition to this, authors conclude that the country and age were the significant factors, while sex was not a significant factor. Oguzoglu [9] identified a decrease in road traffic crashes, fatalities, and injuries during the COVID-19 stay-at-home orders implemented in Turkey. The author concluded that nearly 200 road crashes and 17,600 injuries had been avoided during COVID-19 stay-at-home orders.

Pathak et al. [11] studied the effect of changes in traffic conditions on road safety during the COVID-19 lockdown in Maharashtra, India. The results showed an increase in fatality rates per road crash during the lockdown, while the total number of road crashes reduced drastically. Shaik and Ahmed [19] studied the COVID-19 impact on road traffic crashes, deaths, injuries, and road travel behavior from 281 research articles from the literature. The results showed a decrease in crashes and injuries but increased crash severity. Shimizu et al. [20] assessed the traffic crash rates in Japan from April 2020 to December 2021 as compared with previous years. The yearly change in the traffic crash rate from 2015 to 2019 was estimated, and the traffic crash rate in 2020 and 2021 was predicted. In 2020, the observed vs. expected rates of traffic crashes were lower from April to December 2020, and the rate of a traffic crash in Japan was 30–40% lower in April–May 2020 than would be expected based on trends from previous years. Valent [24] compared the road traffic crashes in Italy during the COVID-19 lockdown period with the year 2019, based on the number of crashes, percent change, nonfatal injuries, fatalities, injury index, and fatality index. Authors concluded that the study witnessed a strong reduction in the number of road traffic crashes in the lockdown period.

### Summary

Many studies have presented the effect of COVID-19 on the road crashes in the lockdown period. These studies are specifically applicable to a respective state or country, a comparison between the 2 countries, and also to multiple countries around the world. It is commonly found that there was a decrease in the total number of crashes and an increase in the crash severity in terms of fatalities in the COVID-19 lockdown period around the world. The reason for this is the less traffic volume which lead to the risky driving behavior of the drivers. In this study, the statistical analysis of the crash data is presented, and the effect of COVID-19 on the road crashes is compared with the same period of previous years as of the lockdown period in 2020. The aim of this study is to access the impact of COVID-19 on the road crashes and comment upon its findings in comparison with the results available in the literature.

## Methods

**Fig. 1:**
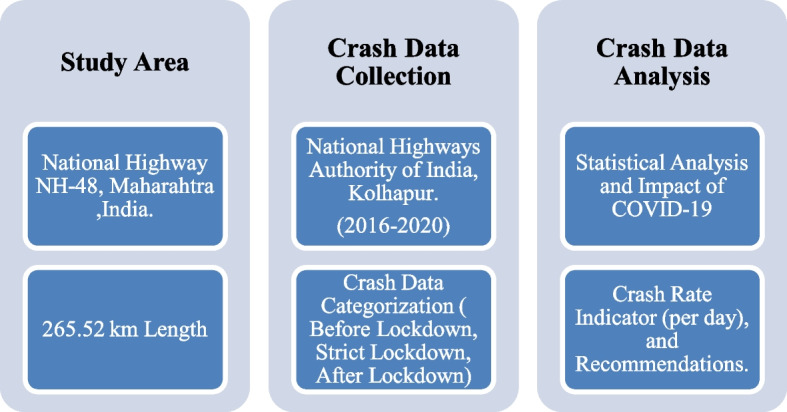
Methodology

The method used to achieve the study objectives includes study area, crash data collection, and crash data analysis as shown in Fig. 1. The detailed methods are discussed in the separate subsections below.

### Study area

National Highway-48 (NH-48) in India starts at “Delhi” and terminates at “Chennai,” traversing through eight states of India and having a length of 2807 km (1744 miles). NH-48 passes through “Delhi, Haryana, Rajasthan, Gujarat, Maharashtra, Karnataka, Andhra Pradesh, and Tamil Nadu.” Its stretch, “Delhi-Mumbai,” was earlier designated NH-8, and “Mumbai-Chennai” was designated NH-4 before all the national highways were renumbered in the year 2010.

The NH-48 passing through the “Maharashtra” state is considered to finalize the study area. In Maharashtra, the highway starts from the “Palghar” district and ends in the “Kolhapur” district. NH-48 has a stretch of 501 km in “Maharashtra” as per the (MoRTH) Ministry of Road Transport and Highways. A section from “Satara” to “Kagal,” having a length of 132.76 km, is finalized as the study area. The total length of the study area is 265.52 km after considering both sides of the section.

### Crash data collection

Crash data from 2016 to 2020 for NH-48 is collected from the “National Highways Authority of India (NHAI), Kolhapur.” There is a total 1614 number of crash records available in the data. The details include chainage-wise location of the crash; date; time; location, i.e., left-hand side (LHS)/right-hand side (RHS) of the highway; vehicle responsible with its vehicle registration number plate; number of affected persons (fatalities, grievous/minor/no injuries/animals killed, if any); time of recording; time of remedial action; and details of the crash. This record is maintained daily and in monthly report format, meaning 60 monthly reports have been collected for 5 years. The 60 monthly crash data reports are merged into a single file in ascending order. Crash data collected from 2016 to 2020 for NH-48 is classified into three separate scenarios, namely, “Before Lockdown,” “After Lockdown,” and “Strict Lockdown.” The scenario is shown in Table 1.

**Table 1:** The scenario of crash data

Sr. No.	Scenario	Date (from-to)	Period (in days)	Period (in months-days)
1	Before lockdown	01 January 2016–24 March 2020	1545	50–24
2	After lockdown	01 June 2020–31 December 2020	214	7–0
3	Strict lockdown	25 March 2020–31 May 2020	68	2–7

### Crash data analysis

The statistical analysis of the crash data over the three scenarios, namely, “Before Lockdown,” “After Lockdown,” and “Strict Lockdown,” is presented in seven different classifications. The crash data is categorized into seven classifications, and the classifications are further subclassified, as shown in Table 2. Each crash has been categorized in a particular classification followed by its sub-classification. The data is used for performing statistical analysis of the crash data of NH-48 across the classification and its sub-classification over the three scenarios. Furthermore, the time-wise analysis and season-wise analysis of the crash data are presented. In the time-wise analysis, 4-time classifications are considered, like 00:00–05:59 AM, 06:00–11:59 AM, 12:00–17:59 PM, and 18:00–23:59 PM. For season-wise analysis, three main classifications of the season are considered, namely, Summer (February to May), Monsoon (June to September), and Winter (October to January). In addition, the impact of COVID-19 on the road crashes for the period 25th March to 31st May every year is discussed, with reference to the lockdown period mentioned by Soni [23] as “Strict Lockdown.”

**Table 2:** Classification of crash data for statistical analysis

Sr. No.	Classification	Sub-classification
1	Nature of crash	Overturning, Head-on collision, Rear-end collision, Collision brush/side wipe, Right angle collision, Skidding, Right-turn collision, and Others
2	Classification of crash	Fatal, Grievous injury, Minor injury, and No injury
3	Causes	Drunken driving, Overspeeding, Vehicle out of control, Fault of driver (any vehicle)/pedestrian, Defect in mechanical condition of road vehicle/road condition, and Not known
4	Road feature	1 lane, 2 lanes, 3 lanes without median, and > 4 lanes with divider
5	Road condition	Straight road, Slight curve, Sharp curve, Flat road, Gentle incline, Steep incline, Hump, and Dip
6	Intersection type and control	T-junction, Y-junction, 4-arm junction, Staggered junction, Junctions with > 4 arms, Roundabout junction, Manned rail crossing, Unmanned rail crossing, and Not known
7	Weather condition	Fine, Mist/fog, Cloudy, Light rain, Heavy rain Hail/sleet, Snow, Strong wind, Dust storm, Very hot, Very cold, and Extraordinary

The three scenarios have different time periods, and hence, the crash frequency cannot be directly compared to comment upon the severity of crashes [17]. Therefore, “Crash Risk Indicator (CRI)” value is estimated per day in all three scenarios. The formula used to calculate the CRI value is as follows:

1 $$\mathrm{CRI}(\mathrm{per}\;\mathrm{day})=(\mathrm{total}\;\mathrm{number}\;\mathrm{of}\;\mathrm{crashes}\;\mathrm{in}\;\mathrm{sub}-\mathrm{classification}/\mathrm{total}\;\mathrm{number}\;\mathrm{of}\;\mathrm{crashes}\;\mathrm{in}\;\mathrm{classification})$$

The Eq. (1) is to be used separately for separate scenario to arrive at CRI value.

Finally, based on the results obtained from the crash data analysis, suitable suggestions are recommended for NH-48 to achieve road safety and road crash reductions.

## Results

**Fig. 2:**
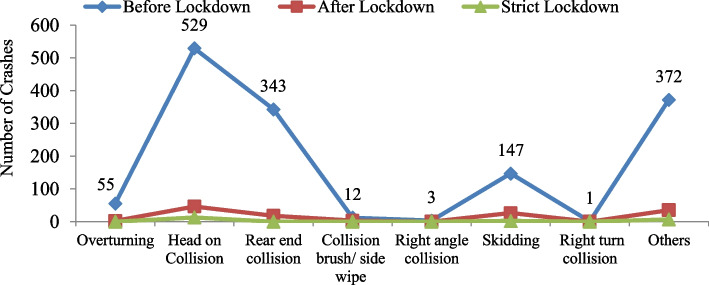
Analysis of crash data by nature of crash

The total number of crashes from 2016 to 2020 is 1614. The crashes which are caused due to various sub-classifications under the “natures of crash” classification are shown in Fig. 2. The data labels visible in Fig. 2 are for the “Before Lockdown” scenario. For the “After Lockdown” scenario, the data labels for Overturning, Head-on collision, Rear-end collision, Collision brush/side wipe, Right angle collision, Skidding, Right-turn collision, and Others are 02, 46, 18, 03, 00, 26, 00, and 35, respectively. For the “Strict Lockdown” scenario, the data labels for Head-on collision, Skidding, and Others are 13, 02, and 07, respectively, and 00 for all the other sub-classifications.

The Crash Rate Indicator (CRI per day) value is calculated and as shown in Table 3. It is clearly seen that the Head-on collision and Others values are increased in the “Strict Lockdown” scenario as compared to “Before Lockdown” scenario. Skidding and Others values are increased in the “After Lockdown” scenario as compared to “Before Lockdown” scenario.

**Table 3:** CRI by nature of crash

Sr. No.	Sub-classifications	Crash rate indicator (CRI per day)		
		**Before Lockdown**	**Strict Lockdown**	**After Lockdown**
1	Overturning	0.038	0.000	0.015
2	Head-on collision	0.362	0.591	0.354
3	Rear-end collision	0.235	0.000	0.138
4	Collision brush/side wipe	0.008	0.000	0.023
5	Right angle collision	0.002	0.000	0.000
6	Skidding	0.101	0.091	0.200
7	Right-turn collision	0.001	0.000	0.000
8	Others	0.254	0.318	0.269

**Fig. 3:**
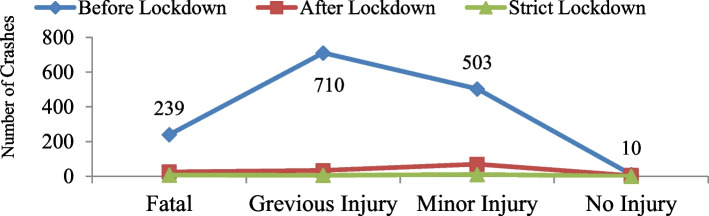
Analysis of crash data by classification of crash

The crashes occurred due to various sub-classifications under the “Classification of crash” classification are shown in Fig. 3. The data labels visible in Fig. 3 are for the “Before Lockdown” scenario. For the “After Lockdown” scenario, the data labels for Fatal, Grievous injury, Minor injury, and No Injury are 25, 33, 69, and 03, respectively. For the “Strict Lockdown” scenario, the data labels for Fatal, Grievous injury, Minor injury, and No injury are 07, 05, 10, and 00, respectively.

The CRI (per day) value is calculated and as shown in Table 4. It is clearly seen that the Fatal and Minor injury values are increased in the “Strict Lockdown” scenario and “After Lockdown” scenario as compared to “Before Lockdown” scenario.

**Table 4:** CRI by classification of crash

Sr. No.	Sub-classifications	Crash rate indicator (CRI per day)		
		**Before Lockdown**	**Strict Lockdown**	**After Lockdown**
1	Fatal	0.163	0.318	0.192
2	Grievous injury	0.486	0.227	0.254
3	Minor injury	0.344	0.455	0.531
4	No injury	0.007	0.000	0.023

**Fig. 4:**
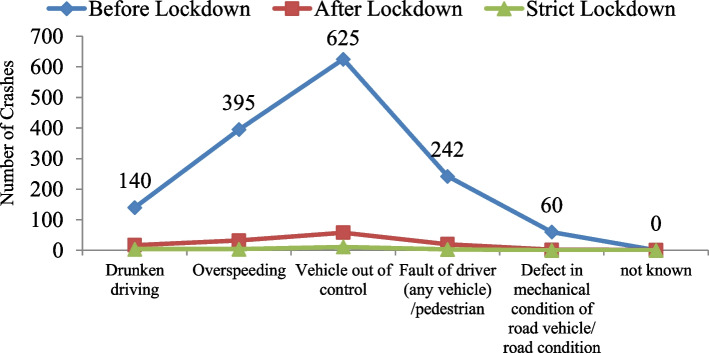
Analysis of crash data by causes of crash

The crashes which are caused due to various sub-classifications under the “causes” classification are shown in Fig. 4. The data labels visible in Fig. 4 are for the “Before Lockdown” scenario. For the “After Lockdown” scenario, the data labels for Drunken driving, Overspeeding, Vehicle out of control, Fault of driver (any vehicle)/pedestrian, Defect in mechanical condition of road vehicle/road condition, and Not known are 17, 32, 58, 20, 02, and 01, respectively. For the “Strict Lockdown” scenario, the data labels for Drunken driving, Overspeeding, Vehicle out of control, Fault of driver (any vehicle)/pedestrian, Defect in mechanical condition of road vehicle/road condition, and Not known are 04, 04, 11, 03, 00, and 00, respectively.

The CRI (per day) value is calculated and as shown in Table 5. It is clearly seen that the Drunken driving and Vehicle out of control values are increased in the “Strict Lockdown” scenario and “After Lockdown” scenario as compared to “Before Lockdown” scenario.

**Table 5:** CRI by causes

Sr. No.	Sub-classifications	Crash rate indicator (CRI per day)		
		**Before Lockdown**	**Strict Lockdown**	**After Lockdown**
1	Drunken driving	0.096	0.182	0.131
2	Overspeeding	0.270	0.182	0.246
3	Vehicle out of control	0.427	0.500	0.446
4	Fault of driver (any vehicle)/pedestrian	0.166	0.136	0.154
5	Defect in mechanical condition of road vehicle/road condition	0.041	0.000	0.015
6	Not known	0.000	0.000	0.008

**Fig. 5:**
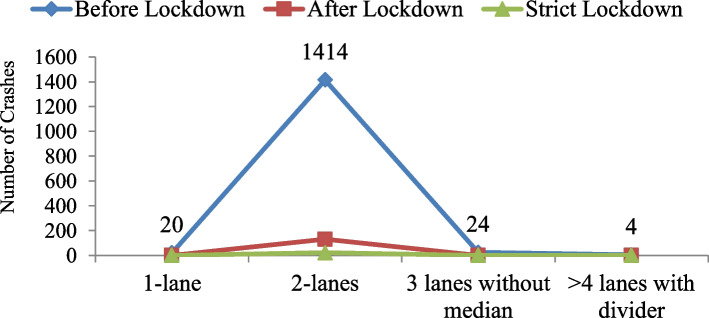
Analysis of crash data by road feature

The crashes which are caused due to various sub-classifications under the “road feature” classification are shown in Fig. 5. The data labels visible in Fig. 5 are for the “Before Lockdown” scenario. For the “After Lockdown” scenario, the data label for 2 lanes is 130 and 00 for all other sub-classifications. For the “Strict Lockdown” scenario, the data label for 2 lanes is 22 and 00 for all other sub-classifications.

The CRI (per day) value is calculated and as shown in Table 6. It is clearly seen that the 2 lanes value is increased in the “Strict Lockdown” scenario and “After Lockdown” scenario as compared to “Before Lockdown” scenario.

**Table 6:** CRI by road feature

Sr. No.	Sub-classifications	Crash rate indicator (CRI per day)		
		**Before Lockdown**	**Strict Lockdown**	**After Lockdown**
1	1 lane	0.014	0.000	0.000
2	2 lanes	0.967	1.000	1.000
3	3 lanes without median	0.016	0.000	0.000
4	> 4 lanes with divider	0.003	0.000	0.000

**Fig. 6:**
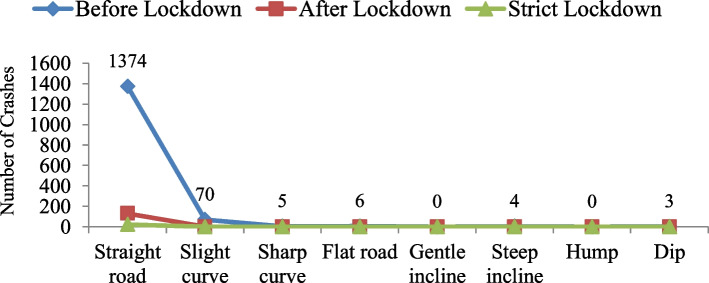
Analysis of crash data by road condition

The crashes which are caused due to various sub-classifications under the “road condition” classification are shown in Fig. 6. The data labels visible in Fig. 6 are for the “Before Lockdown” scenario. For the “After Lockdown” scenario, the data label for Straight road is 130 and 00 for all other sub-classifications. For the “Strict Lockdown” scenario, the data label for Straight road is 22 and 00 for all other sub-classifications.

The CRI (per day) value is calculated and as shown in Table 7. It is clearly seen that the Straight road value is increased in the “Strict Lockdown” scenario and “After Lockdown” scenario as compared to “Before Lockdown” scenario.

**Table 7:** CRI by road condition

Sr. No	Sub-classifications	Crash rate indicator (CRI per day)		
		**Before Lockdown**	**Strict Lockdown**	**After Lockdown**
1	Straight road	0.940	1.000	1.000
2	Slight curve	0.048	0.000	0.000
3	Sharp curve	0.003	0.000	0.000
4	Flat road	0.004	0.000	0.000
5	Gentle incline	0.000	0.000	0.000
6	Steep incline	0.003	0.000	0.000
7	Hump	0.000	0.000	0.000
8	Dip	0.002	0.000	0.000

**Fig. 7:**
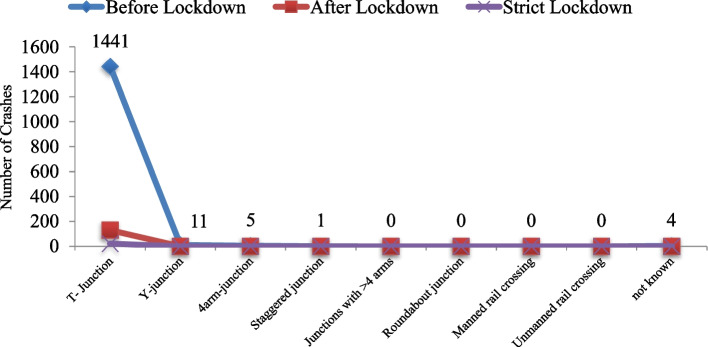
Analysis of crash data by intersection type and control

The crashes which are caused due to various sub-classifications under the “intersection type and control” classification are shown in Fig. 7. The data labels visible in Fig. 7 are for the “Before Lockdown” scenario. For the “After Lockdown” scenario, the data label for T-junction is 130 and 00 for all other sub-classifications. For the “Strict Lockdown” scenario, the data label for T-junction is 22 and 00 for all other sub-classifications.

The CRI (per day) value is calculated and as shown in Table 8. It is clearly seen that the T-junction value is increased in the “Strict Lockdown” scenario and “After Lockdown” scenario as compared to “Before Lockdown” scenario.

**Table 8:** CRI by intersection type and control

Sr. No.	Sub-classifications	Crash Rrate indicator (CRI per day)		
		**Before Lockdown**	**Strict Lockdown**	**After Lockdown**
1	T-junction	0.986	1.000	1.000
2	Y-junction	0.008	0.000	0.000
3	4-arm junction	0.003	0.000	0.000
4	Staggered junction	0.001	0.000	0.000
5	Junctions with > 4 arms	0.000	0.000	0.000
6	Roundabout junction	0.000	0.000	0.000
7	Manned rail crossing	0.000	0.000	0.000
8	Unmanned rail crossing	0.000	0.000	0.000
9	Not known	0.003	0.000	0.000

**Fig. 8:**
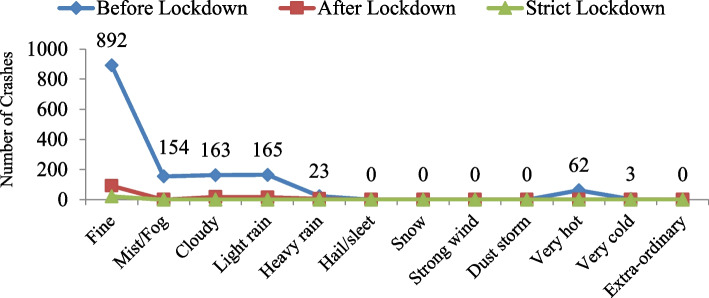
Analysis of crash data by weather condition

The crashes which are caused due to various sub-classifications under the “weather condition” classification are shown in Fig. 8. The data labels visible in Fig. 8 are for the “Before Lockdown” scenario. For the “After Lockdown” scenario, the data labels for Fine, Cloudy, Light rain, and Heavy rain are 92, 18, 15, 05, and 00 for all other sub-classifications. For the “Strict Lockdown” scenario, the data label for Fine is 22 and 00 for all other sub-classifications.

The CRI (per day) value is calculated and as shown in Table 9. It is clearly seen that the Fine value is increased in the “Strict Lockdown” scenario and “After Lockdown” scenario as compared to “Before Lockdown” scenario. Cloudy, Light rain and Heavy rain values are increased in the “After Lockdown” scenario as compared to “Before Lockdown” scenario.

**Table 9:** CRI by weather condition

Sr. No.	Sub-classifications	Crash Rate Indicator (CRI per day)		
		**Before Lockdown**	**Strict Lockdown**	**After Lockdown**
1	Fine	0.610	1.000	0.708
2	Mist/fog	0.105	0.000	0.000
3	Cloudy	0.111	0.000	0.138
4	Light rain	0.113	0.000	0.115
5	Heavy rain	0.016	0.000	0.038
6	Hail/sleet	0.000	0.000	0.000
7	Snow	0.000	0.000	0.000
8	Strong wind	0.000	0.000	0.000
9	Dust storm	0.000	0.000	0.000
10	Very hot	0.042	0.000	0.000
11	Very cold	0.002	0.000	0.000
12	Extra-ordinary	0.000	0.000	0.000

### Time-wise analysis

**Fig. 9:**
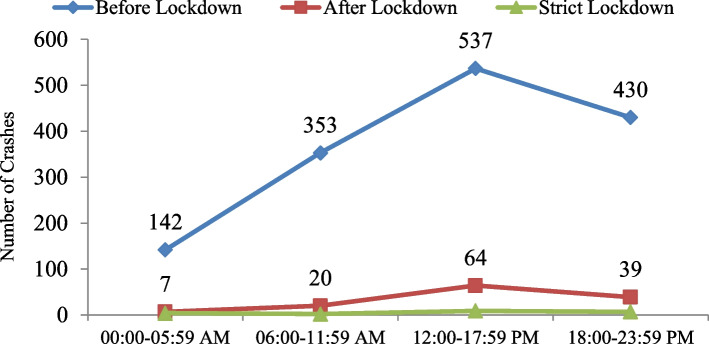
Time-wise analysis of crash data

The time-wise analysis of crash data is shown in Fig. 9. The data labels for the “Before Lockdown” scenario and the “After Lockdown” scenario are shown in Fig. 9. In the case of the “Strict Lockdown” scenario of time-wise analysis, the data labels for 00:00–05:59 AM, 06:00–11:59 AM, 12:00–17:59 PM, and 18:00–23:59 PM are 04, 02, 09, and 07, respectively.

The CRI (per day) value is calculated and is shown in Table 10. It is clearly seen that the values of  12:00–17:59 PM and 18:00–23:59 PM are increased in the “Strict Lockdown” scenario and “After Lockdown” scenario as compared to “Before Lockdown” scenario, whereas the 00:00–05:59 AM value is increased in the “Strict Lockdown” scenario as compared to “Before Lockdown” scenario.

**Table 10:** CRI by time-wise analysis

Sr. No.	Sub-classifications	Crash Rate Indicator (CRI per day)		
		**Before Lockdown**	**Strict Lockdown**	**After Lockdown**
1	00:00–05:59 AM	0.097	0.182	0.054
2	06:00–11:59 AM	0.241	0.091	0.154
3	12:00–17:59 PM	0.367	0.409	0.492
4	18:00–23:59 PM	0.294	0.318	0.300

### Season-wise analysis

**Fig. 10:**
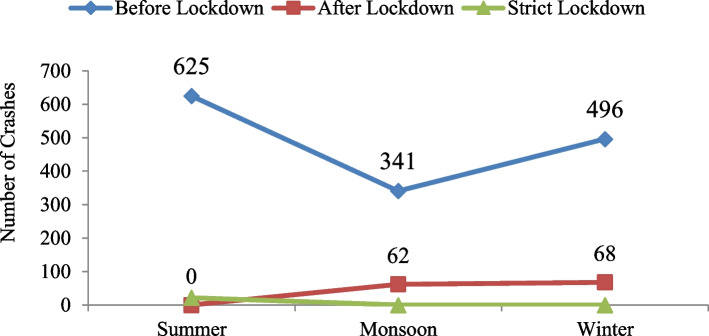
Season-wise analysis of crash data

The season-wise analysis of crash data is also shown in Fig. 10. The data labels for the “Before Lockdown” scenario and the “After Lockdown” scenario are shown in Fig. 10. In the case of the “Strict Lockdown” scenario of season-wise analysis, the data labels for Summer (February to May), Monsoon (June to September), and Winter (October to January) are 22, 00, and 00, respectively.

The CRI (per day) value is calculated and is shown in Table 11. It is clearly seen that the value of Summer is increased in the “Strict Lockdown” scenario as compared to “Before Lockdown” scenario, whereas the Monsoon and Winter values are increased in the “After Lockdown” scenario as compared to “Before Lockdown” scenario.

**Table 11:** CRI by season-wise analysis

Sr. No.	Subclassifications	Crash Rate Indicator (CRI per day)		
		**Before Lockdown**	**Strict Lockdown**	**After Lockdown**
1	Summer	0.427	1.000	0.000
2	Monsoon	0.233	0.000	0.477
3	Winter	0.339	0.000	0.523

### COVID-19 impact

The lockdown due to COVID-19 in India was declared from 25 March 2020 to 31 May 2020. The “Strict Lockdown” period in 2020 was from “25 March 2020 to 3 May 2020”; therefore, a comparison with the previous 4 years of the same period is studied. In the period from “25 March 2019 to 31 May 2019” and “25 March 2018 to 31 May 2018,” the total number of crashes was 56 and 56, respectively. In the period from “25 March 2017 to 31 May 2017,” the total number of crashes was 16, whereas from “25 March 2016 to 31 May 2016,” it was 28.

The detailed classification and sub-classification wise data of the COVID-19 impact in the previous 4 years is shown in Table 12. “NA” stands for “not applicable” in Table 12, and 1 to 12 sub-classifications represent the individual sub-classifications for the classification in the sequential order. For example, in classification by nature of crash, there are 8 sub-classifications as shown in Table 2. Now, 1 represents the first sub-classification which is Overturning, 2 represents second sub-classification which is Head-on collision, 3 represents the third sub-classification which is rear-end collision etc. The use of 1 to 12 sub-classifications in Table 12 is for the amalgamation of classification-wise and sub-classification wise data in a single table. The sequence followed for the 1 to 12 classifications is as per the individual classifications sub-classification that appeared on the *X*-axis of Figs. 2, 3, 4, 5, 6, 7 and 8.

**Table 12:** The impact of COVID-19 on the previous 4 years

Sr. No.	Classification (type)	Year (25th March to 31st May)	Sub-classification											
			**1**	**2**	**3**	**4**	**5**	**6**	**7**	**8**	**9**	**10**	**11**	**12**
**1**	B (nature of crash)	2016	2	6	11	0	0	0	0	9	NA	NA	NA	NA
		2017	1	4	7	0	0	0	0	4	NA	NA	NA	NA
		2018	0	26	3	0	0	11	0	16	NA	NA	NA	NA
		2019	0	26	0	0	0	10	0	20	NA	NA	NA	NA
		2020-COVID-19	0	13	0	0	0	2	0	7	NA	NA	NA	NA
**2**	C (Classification of crash)	2016	4	19	5	0	NA	NA	NA	NA	NA	NA	NA	NA
		2017	5	5	6	0	NA	NA	NA	NA	NA	NA	NA	NA
		2018	7	19	28	2	NA	NA	NA	NA	NA	NA	NA	NA
		2019	4	15	37	0	NA	NA	NA	NA	NA	NA	NA	NA
		2020-COVID-19	7	5	10	0	NA	NA	NA	NA	NA	NA	NA	NA
**3**	D (causes)	2016	1	5	14	8	0	0	NA	NA	NA	NA	NA	NA
		2017	0	9	4	1	2	0	NA	NA	NA	NA	NA	NA
		2018	5	17	27	4	3	0	NA	NA	NA	NA	NA	NA
		2019	5	10	31	10	0	0	NA	NA	NA	NA	NA	NA
		2020-COVID-19	4	4	11	3	0	0	NA	NA	NA	NA	NA	NA
**4**	E (road feature)	2016	2	26	0	0	NA	NA	NA	NA	NA	NA	NA	NA
		2017	0	16	0	0	NA	NA	NA	NA	NA	NA	NA	NA
		2018	0	56	0	0	NA	NA	NA	NA	NA	NA	NA	NA
		2019	2	53	1	0	NA	NA	NA	NA	NA	NA	NA	NA
		2020-COVID-19	0	22	0	0	NA	NA	NA	NA	NA	NA	NA	NA
**5**	F (road condition)	2016	22	6	0	0	0	0	0	0	NA	NA	NA	NA
		2017	15	0	0	1	0	0	0	0	NA	NA	NA	NA
		2018	56	0	0	0	0	0	0	0	NA	NA	NA	NA
		2019	56	0	0	0	0	0	0	0	NA	NA	NA	NA
		2020-COVID-19	11	0	0	0	0	0	0	0	NA	NA	NA	NA
**6**	G (intersection type & control)	2016	28	0	0	0	0	0	0	0	0	NA	NA	NA
		2017	16	0	0	0	0	0	0	0	0	NA	NA	NA
		2018	56	0	0	0	0	0	0	0	0	NA	NA	NA
		2019	49	3	0	0	0	0	0	0	4	NA	NA	NA
		2020-COVID-19	22	0	0	0	0	0	0	0	0	NA	NA	NA
**7**	H (weather condition)	2016	8	3	6	5	2	0	0	0	0	1	3	0
		2017	5	3	5	1	0	0	0	0	0	2	0	0
		2018	55	0	0	1	0	0	0	0	0	0	0	0
		2019	56	0	0	0	0	0	0	0	0	0	0	0
		2020-COVID-19	22	0	0	0	0	0	0	0	0	0	0	0

## Discussion

The pattern of factors responsible for crashes in all three scenarios is almost parallel; therefore, the opportunities are discussed in general for all scenarios to improve the current road safety of the study area.

### Opportunities and challenges

The entire study area must have more lanes on either side of the highway since almost all the road crashes were witnessed on “2 lanes.” The straight roads must be provided with clearly visible road markings and rumble strips near the junctions, as the majority of road crashes were witnessed on “Straight road” and “T junction.” There has to be strict traffic police patrolling for the vehicles coming in the opposite direction through the shoulders to avoid significant crashes due to “Head-on collision.” Implementation of a speed limit and its monitoring is essential as the crashes were due to “Vehicle out of control” and “Overspeeding.” These crashes majorly result in “Fatal,” “Grievious injury,” and “Minor injury.” No crashes are subclassified under “No injury” after a crash; therefore, the severity of the crashes must be understood while implementing the recommendations.

More than 60% of road crashes occur in “Fine” weather conditions, so “Weather condition” is not the primary classification responsible for crashes. In addition, 23% of crashes were witnessed in the “Monsoon” season, the lowest of all, which implies that people are aware of driving safely in the “Monsoon” season. Also, less than 10% of crashes occurred between “00:00 and 05:59 AM,” implying that midnight driving is safe, whereas day driving accounts for 54% of crashes, which is very serious.

More than 25% of crashes occurred due to “Others” reason in the nature of the crash, which is to be identified and included in the subclassification. The number of crashes under this subclassification is almost 1/4, and it becomes mysterious to unfold the causes and give suitable recommendations based on the current crash data. The decision-makers should understand and implement these recommendations as the fatalities in 2020 were found to increase despite the COVID-19 “Strict Lockdown” scenario. The methodology used in this study can be used for any study having the crash data which covers Before Lockdown, Strict Lockdown, and After Lockdown time period. This type of study should be worked out on all the highways around the world, especially in low- and middle-income countries, on priority to understand road safety performance.

### Comparison of results with other studies

The results of the present study area due to the impact of COVID-19 are discussed first. Comparing crashes that occurred in 2020 due to COVID-19 with years 2016, 2017, 2018, and 2019 revealed the following information. The crashes that occurred under the “Classification of crash” classification with its subclassification as “Grievous injury” and the crashes under the “road condition” classification with its sub-classification as “Straight road” are reduced as compared to all the previous 4 years. Surprisingly, the crashes that occurred under the “nature of crash” classification with its sub-classification as “Right angle collision” and the crashes under the “Classification of crash” classification with its sub-classification as “Fatal” are increased as compared to all previous 4 years. The crashes occurred under the “nature of crash” classification with its sub-classifications as “Head-on collision,” “Skidding,” “Others,” the crashes under the “Classification of crash” classification with its sub-classification as “minor injury,” the crashes under the “causes” classification with its sub-classifications as “Drunken driving,” “Overspeeding,” “Vehicle out of control,” “Fault of driver (any vehicle)/pedestrian,” the crashes under the “Road feature” classification with its sub-classification as “2-lanes,” the crashes under the “intersection type & control” with its sub-classification as “T-junction” the crashes under the “Weather condition” classification with its sub-classification as “Fine,” were 50% reduced and 50% increased as compared to all previous 4 years. The remaining all classifications with their sub-classifications have shown a “0” number of crashes in 2020 due to the COVID-19 impact.

The other studies around the world concluded in general that the number of crashes due to the impact of COVID-19 is reduced, whereas the severity of the crashes is increased. The crash data, period of comparison, and study area were different for every individual study. In this study, a detailed analysis over classifications and its sub-classifications is considered. Therefore, the results revealed that the number of crashes is reduced in some of the sub-classifications and increased in some of the sub-classifications. The crash severity is increased in terms of fatalities, whereas decreased in terms of Grievious injuries. This means a mixed impact on the number of crashes as well as on the crash severity is observed.

## Conclusions

The aim of the study is to analyze the crash data in three scenarios according to the classification and sub-classifications available in the crash data; see the impact of COVID-19 on road crashes and give recommendations based on the results for improving the road safety. The available crash data, its categorization in three scenarios, and calculating the Crash Risk Indicator values helped to understand which points are more critical for the road safety and that helped to arrive at the suitable recommendations to improve the study area. There were 1462 crashes witnessed in the “Before Lockdown” scenario, 130 crashes in the “After Lockdown” scenario, and 22 in the “Strict Lockdown” scenario. The most important parameters contributing to the crashes are discussed separately under each scenario, along with the impact of COVID-19.

### Before Lockdown

The “Head-on collision” accounts for 529 (36.18%) crashes, and 372 (25.38%) crashes were identified as “Others.” Also, “rear-end collision” accounts for 343 (23.46%) crashes under the classification of nature of crash. Seven-hundred ten (48.56%) crashes resulted in “Grievous injury,” 239 (16.30%) crashes were “Fatal,” and 503 (34.40%) crashes were “Minor-injury.” Under the classification of causes, 625 (42.75%) crashes were due to “Vehicle out of control,” and 395 (27.02%) crashes were due to “Overspeeding.” In the case of the classification under road features, road conditions, and intersection type and control, 1414 (96.72%) crashes occurred on “2 lanes,” 1374 (93.98%) crashes occurred on “Straight road,” and 1441 (98.56%) crashes were witnessed on “T-junction,” respectively. Under the classification of weather conditions, 892 (60.85%) crashes happened in “Fine” weather condition, whereas 165 (11.26%) crashes occurred in “Light rain.”

In the case of time-wise analysis, 537 (36.73%) crashes occurred between “12:00 and 17:59 PM,” 430 (29.41%) crashes occurred between “18:00 and 23:59 PM,” and 353 (24.08%) crashes happened between “06:00 and 11:59 AM.” There were 625 (42.63%) crashes in the “Summer” season, 496 (33.93%) crashes in “Winter,” and 341 (23.26%) crashes occurred in the “Monsoon” season.

### After Lockdown

The “Head-on collision” accounts for 46 (35.38%) crashes, and 35 (26.92%) crashes were identified as “Others” under the classification of nature of crash. Also, “Rear-end collision” accounts for 18 (13.85%) crashes. Thirty-three (25.38%) crashes resulted in “Grievous injury,” 25 (19.23%) crashes were “Fatal,” and 69 (53.08%) crashes were “Minor injury.” Under the classification of causes, 58 (44.62%) crashes were due to “Vehicle out of control,” and 32 (24.62%) crashes were due to “Overspeeding.” In the case of the classification under road features, road conditions, and intersection type and control, 130 (100.00%) crashes occurred on “2 lanes,” 130 (100.00%) crashes occurred on “Straight road,” and 130 (100.00%) crashes were witnessed on “T-junction,” respectively. Under the classification of weather conditions, 92 (70.77%) crashes happened in “Fine” weather condition, whereas 18 (13.85%) crashes occurred in “Cloudy.”

In the case of time-wise analysis, 64 (49.23%) crashes occurred between “12:00 and 17:59 PM,” 39 (30.00%) crashes occurred between “18:00 and 23:59 PM,” and 20 (15.38%) crashes happened between “06:00 and 11:59 AM.” There were 68 (52.31%) crashes that took place in the “Winter” season, and 62 (47.69%) crashes in the “Monsoon” season.

### Strict Lockdown

The “Head-on collision” holds 13 (59.10%) crashes, and there are 07 (31.82%) crashes that were identified as “Others” under the classification of nature of crash. Five (22.73%) crashes resulted in “Grievous injury,” 7 (31.82%) crashes were “Fatal,” and 10 (45.45%) crashes were “Minor injury.” Under the classification of causes, 11 (50.00%) crashes were due to “Vehicle out of control,” and 4 (18.18%) crashes were due to “Overspeeding.” In the case of the classification under road features, road conditions, and intersection type and control, 22 (100.00%) crashes occurred on “2 lanes,” 22 (100.00%) crashes occurred on “Straight road,” and 22 (100.00%) crashes were witnessed on “T-junction,” respectively. Under the weather conditions classification, 22 (100.00%) crashes occurred in “Fine” weather conditions. In the case of time-wise analysis, 9 (40.91%) crashes occurred between “12:00 and 17:59 PM,” 7 (31.82%) crashes occurred between “18:00 and 23:59 PM,” and 4 (18.18%) crashes occurred between “00:00 and 05:59 AM.” twenty-two (100.00%) crashes were witnessed in the “Summer” season.

### Impact of COVID-19

There were 22 crashes in the “Strict Lockdown” scenario, and the comparison of the same period in previous years is discussed in the “Results” section. Now, considering the number of COVID-19 crashes in 2020 as the baseline, the impact of COVID-19 on the previous years of crashes reveals critical information. The crashes in the COVID-19 “Strict Lockdown” scenario witnessed a fall of 254.55% compared to 2019 and 2018. Surprisingly, there was a rise of 137.5% and a fall of 127.27% in crashes of the COVID-19 2020 “Strict Lockdown” scenario, compared to 2017 and 2016, respectively.

In addition to these, the study calculates the Crash Risk Indicator (CRI) value per day for all the three scenarios. This helps to identify the key sub-classifications which are at high impact level for road safety. The sub-classifications like Others, Fatal, Minor injury, Drunken driving, Vehicle out of control, 2 lanes, Straight road, T-junction, Fine, 12:00–17:59 PM, 18:00–23:59 PM, and Summer are observed to have increased CRI values in “Strict Lockdown” as well as “After Lockdown” scenario as compared to “Before Lockdown” scenario. The sub-classifications like Skidding, Cloudy, Light rain, Heavy rain, 00:00–05:59 AM, Monsoon, and Winter are observed to have increased CRI values in “After Lockdown” scenario as compared to “Before Lockdown” scenario, whereas the sub-classification like Head-on collision is observed increasing in the “Strict Lockdown” scenario as compared to “Before Lockdown” scenario.

## Data Availability

Data is confidential and can be made available upon request.

## Abbreviations

COVID-19: Coronavirus disease
IRTAD: International Road Traffic and Accident Database
PIN: Performance index
NH: National highway
AP: Andhra Pradesh
USA: United States of America
MoRTH: Ministry of Road Transport and Highways
km: Kilometers
NHAI: National Highways Authority of India
LHS: Left-hand side
RHS: Right-hand side
AM: Anti meridiem
PM: Post meridiem
NA: Not applicable
